# Malondialdehyde-acetaldehyde (MAA) adducted surfactant protein induced lung inflammation is mediated through scavenger receptor a (SR-A1)

**DOI:** 10.1186/s12931-017-0517-x

**Published:** 2017-02-13

**Authors:** Muna Sapkota, Jane M. DeVasure, Kusum K. Kharbanda, Todd A. Wyatt

**Affiliations:** 1 Department of Environmental, Agricultural and Occupational Health, College of Public Health, Nebraska Medical Center, University of Nebraska Medical Center, 985910, Omaha, NE 68198-5910 USA; 2 Department of Internal Medicine, Division of Pulmonary, Critical Care, Sleep and Allergy, University of Nebraska Medical Center, Omaha, NE USA; 3 Department of Internal Medicine, Division of Gastroenterology, University of Nebraska Medical Center, Omaha, NE USA; 4 VA Nebraska-Western Iowa Health Care System, Omaha, NE USA

**Keywords:** Lung, MAA adduct, Scavenger receptor A, Inflammation

## Abstract

**Background:**

Co-exposure to cigarette smoke and alcohol leads to the generation of high concentrations of acetaldehyde and malondialdehyde in the lung. These aldehydes being highly electrophilic in nature react with biologically relevant proteins such as surfactant protein D (SPD) through a Schiff base reaction to generate SPD adducted malondialdehyde-acetaldehyde adduct (SPD-MAA) in mouse lung. SPD-MAA results in an increase in lung pro-inflammatory chemokine, keratinocyte chemoattractant (KC), and the recruitment of lung lavage neutrophils. Previous *in vitro* studies in bronchial epithelial cells and macrophages show that scavenger receptor A (SR-A1/CD204) is a major receptor for SPD-MAA. No studies have yet examined the *in vivo* role of SR-A1 in MAA-mediated lung inflammation. Therefore, we hypothesize that in the absence of SR-A1, MAA-induced inflammation in the lung is reduced or diminished.

**Methods:**

To test this hypothesis, C57BL/6 WT and SR-A1 KO mice were nasally instilled with 50 μg/mL of SPD-MAA for 3 weeks (wks). After 3 weeks, bronchoalveolar lavage (BAL) fluid was collected and assayed for a total cell count, a differential cell count and CXCL1 (KC) chemokine. Lung tissue sections were stained with hematoxylin and eosin (H&E) and antibodies to MAA adduct.

**Results:**

Results showed that BAL cellularity and influx of neutrophils were decreased in SR-A1 KO mice as compared to WT following repetitive SPD-MAA exposure. MAA adduct staining in the lung epithelium was decreased in SR-A1 KO mice. In comparison to WT, no increase in CXCL1 was observed in BAL fluid from SR-A1 KO mice over time.

**Conclusions:**

Overall, the data demonstrate that SR-A1/CD204 plays an important role in SPD-MAA induced inflammation in lung.

## Background

People who abuse alcohol are likely to smoke cigarettes, and similarly, smokers are more likely to drink alcohol than non-smokers [1]. Among individuals with alcohol use disorders (AUDs), smoking rates are estimated to be 90% and more than 70% of these individuals smoke at least one pack of cigarettes per day [2, 3]. Well established as the major cause of all lung cancers, cigarette smoking is also a risk factor for respiratory tract infections like pneumonia and tuberculosis. Smoking is also the primary risk factor for chronic obstructive pulmonary disease (COPD) [4], projected to be the third leading global cause of death by 2030 [5]. Because the chronic consumption of alcohol has a wide range of effects on lung function, it could increase the risks of pneumonia, acute respiratory distress syndrome (ARDS) [6] and COPD [7].

Although most ingested alcohol is metabolized in the liver, significant concentrations of ethanol reach the airway passages via the bronchial circulation [8]. In the lung of chronic alcohol drinkers, airways can be continuously exposed to high concentrations of alcohol due to the “recycling” of alcohol vapor [9]. Generally, alcohol is metabolized by the alcohol dehydrogenase (ADH) pathway to generate acetaldehyde [8]. In chronic alcohol consumption, however, the CYP2E1 pathway is induced, leading to oxidative stress, lipid peroxidation and generation of malondialdehyde [10, 11]. In alcohol abusers who smoke cigarettes, even higher concentrations of reactive aldehydes are formed in the lung. Metabolism of alcohol through ADH and CYP2E1 leads to generation of acetaldehyde (AA) and malondialdehyde (MDA) [12]. In addition, pyrolysis of tobacco generates high concentrations of AA [13]. Cigarette smoke also induces oxidative stress, leading to lipid peroxidation resulting in high concentrations of MDA [14]. These aldehydes in lung, being highly reactive and electrophilic, bind to nucleophilic sites on proteins through a Schiff base reaction [15], leading to the formation of hybrid protein adducts. Unlike other individual aldehyde protein adducts, this hybrid malondialdehyde-acetaldehyde (MAA) adduct is very stable [16]. The lungs of alcohol abusers who also smoke cigarettes are the ideal environment for the formation of MAA adduct [17]. Lung surfactant protein D (SPD), produced by type II alveolar epithelial cells, is one target protein adducted by reactive aldehydes to form SPD-MAA [17]. MAA adducts in lung have been shown to delay wound healing and increase protein kinase C-dependent IL-8 release as well as decrease cilia beating frequency [18, 19]. Additionally, MAA adducted protein instilled into lungs of mice produce inflammatory injury [20].

Scavenger receptor A (SR-A1/CD204) expression is mainly confined to macrophages but is also present on dendritic cells, endothelial cells, Kupffer cells, airway epithelial cells and vascular smooth muscle cells [21, 22]. SR-A1 plays an important, well-established role in atherosclerosis [23–25]. SR-A1 can bind to a broad range of ligands such as oxidized LDL, acetylated LDL, fucoidan, dextran sulfate and modified self proteins [26] as well as a number of conserved microbial structures, such as bacterial lipopolysaccharide and lipoteichoic acid [27]. Based upon *in vitro* evidence, SR-A1 may play an important role in innate immunity, and an affinity for modified lipids and pathogens might suggest its role in inflammation [28]. SR-A1 has been shown to be important in the uptake of the MAA adduct in bronchial epithelial cells [29] and liver sinusoidal endothelial cells [30]. Absence of this receptor has been shown to reduce IL-8 release from mouse tracheal epithelial cells [29] as well as decrease antibody response to malondialdehyde acetaldehyde albumin (MAA-Alb) [30]. SR-A1 has also been shown to be one of the primary receptors for MAA adduct on macrophages [31].

Our previous study in mice shows that MAA has a pro-inflammatory effect in lung after 3 weeks of instillation as an increase in the pro-inflammatory chemokine, keratinocyte chemoattractant (KC; CXCL1), and lung lavage neutrophils was observed [20]. In both bronchial epithelial cells and macrophages, SR-A1 primarily binds MAA adducts [29, 31] and plays an important role initiating MAA-mediated effects. However, the role of SR-A1 in MAA adduct-mediated lung inflammation and injury has not yet been studied, therefore we hypothesized that SR-A1 has an important role in MAA-mediated lung inflammation, and that in the absence of this receptor, the effects of MAA in mouse lung is decreased. We show, for the first time, the role of SR-A1 in MAA-mediated lung inflammatory effects in a mouse model.

## Methods

### Mice

WT C57BL/6 mice were purchased from the Charles River (Wilmington, MA) at 6–8 weeks of age and SR-A1 (CD204) knockout mice on C57BL/6 background were bred from homozygous SR-A1-deficient mice (−/−) (B6.Cg-*Msr1* ^*tm1Csk*^/J; Jackson Laboratory, Bar Harbor, ME). All mice were housed in group cages and received standard rodent chow and water *ad libitum* for 1 week before the start of the experiment. Mice were monitored daily and weighed weekly. All experimental protocols were reviewed and approved by the Institutional Animal Care and Use Committee of the University of Nebraska Medical Center (protocol number 04-059-08).

### Preparation of MAA adducted protein

Human surfactant protein D (SPD) adducted to MAA (SPD-MAA) was prepared as previously reported [20]. Briefly, approximately 1–1.5 mg/mL of SPD was incubated with 1.0 mM acetaldehyde and 1.0 mM MDA in pyrogen-free PBS. The pH was brought to 7.4, and maintained at 37 °C for 72 h. At the end of incubation, the reaction mixture was exhaustively dialyzed against pyrogen-free phosphate buffer solution for 24 h at 4 °C. The endotoxin level in the MAA-SPD was measured by limulus assay and was below the limit of detection.

### Intranasal instillation

The intranasal instillation was performed as previously described [20]. Briefly, mice were first assigned to three treatment groups: saline, SPD and SPD-MAA. Non-adducted protein control (SPD) was used to rule out any potential immunological side effects. All three treatments (saline, SPD-MAA and SPD) were sterile and free of endotoxin. For intranasal instillation each mouse was anesthetized using isoflurane inhalation and then positioned with its head held back to make the nasal pathway vertical. 50 μL of the treatment solution (sterile saline or 50 μg/mL of SPD-MAA or SPD) was gently placed on the nasal openings with a pipette tip. After the instillation, each mouse was held in the same position to ensure complete inhalation and then monitored until it was awake and moving around normally. This procedure was done one time and also repeated daily for 1–3 weeks. None of the mice showed any sign of distress.

### Bronchoalveolar lavage (BAL)

After the instillation period, mice were euthanized by isoflurane overdose. The trachea was then exposed and a cannula inserted just below the larynx. The proximal end of the trachea was tied around the cannula and 1.0 mL of sterile PBS (Gibco, Grand Island, NY, USA) was instilled into the lungs and recovered by aspiration three separate times. The BAL fluid was centrifuged at 250 *g* to collect cells. The supernatant from the first pull was stored at −80 °C for later analysis of cytokines/chemokines. Cells from the 3 ml were resuspended in PBS, counted using a hemocytometer and then cytospun (Cytopro Cytocentrifuge, Wescor Inc. Logan, UT, USA) onto slides. The slides were stained with Hema three stain set (Fisher, Kalamazoo, MI). Later, cell differential counts were carried out on the slides with a minimum of 200 cells per slide counted for differential analysis per mouse.

### Lung histology

After whole lung lavage, the lung from each treatment group was tied with a suture thread via the trachea to the cannula. Once tied, the lung was slowly removed from the thoracic cavity. Then the lungs were slowly inflated with 1 mL of 10% formalin (Sigma, St. Louis, MO). The lungs were hung under a pressure of 15 cmH_2_O for 24 h while submerged in 10% formalin to obtain uniform lung inflation during fixation. Subsequently, the lung tissues were arranged in cassettes and send to a tissue processing facility where the lung tissue were dehydrated and embedded in paraffin. 5 μm sections were made from lung tissue. The sections were stained for hematoxylin and eosin (H&E) or utilized later for immunohistochemistry. Each slide was reviewed at scanning magnifications (×2, ×4, and ×10 objectives; Nikon Eclipse model E600 microscope). The slides were reviewed to assess the influx of inflammatory cells (mostly neutrophils).

### Immunohistochemistry

Immunohistochemical staining of the lung tissue section was performed as previously reported [31] with slight modification. Formalin-fixed, paraffin-embedded sections of 5 μm thick tissue were deparaffinized through Safeclear II™ tissue clearing agent (Fisher) and later rehydrated using a graded series of alcohol washes (100, 95, 80, 50% ethanol). The slides were then rinsed three times in PBS. A heat-induced epitope retrieval method was performed for antigen unmasking. Briefly, slides were immersed in preheated antigen retrieval solution (DIVA Decloaker solution; Biocare Medical, Concord, CA) and steamed for 20 min at 95 °C in a vegetable steamer. After heating, the slides were allowed to cool and then rinsed with PBS for three times. After washing, the slides were incubated with 0.1% Triton in PBS for another 10 min. The slides were then washed with PBS for three times for 5 min each. Endogenous peroxidase activity was quenched with 3% hydrogen peroxide in methanol for 15 min. After being washed three times in PBS, slides were blocked with non-fat milk (5%) in PBS-tween (0.1%) for another 2 h in a humidity chamber at RT. Slides were incubated overnight with primary antibody and respective isotype control in a humidity chamber: rabbit anti-MAA and rabbit-IgG (dilution 1:1000; Abcam, Inc., Cambridge, MA). After being washed, slides were incubated with the appropriate HRP conjugated goat-anti-rabbit IgG (dilution 1:1000; Jackson ImmunoResearch Laboratories, Inc., Grand Island, NY) secondary antibody in a humidity chamber. After 1 h, slides were rinsed and developed with Chromogen substrate (IMMPACT DAB, Vector, Burlingame, CA) followed by counter staining with 1% Meyer’s hematoxylin. Finally the slides were dehydrated through a series of ethanols and fixed with Safeclear II™ tissue clearing agent.

### Chemokine Assay

Murine, keratinocyte chemoattractant (KC; CXCL1) level was determined in BAL fluid according to the manufacturer's instructions using a commercially available ELISA kit (R&D Systems, Minneapolis, MN).

### Lung slice protocol

Lung slices were prepared as previously described [20]. Briefly, C57BL/6 mice, between 7 and 9 weeks old, were sacrificed by isoflurane overdose. Lungs were allowed to deflate after which a syringe filled with a warm (37 °C) solution of 2% agarose (type VII or VII-A: low gelling temperature; Invitrogen, Carlsbad, CA, USA) in Hanks Balanced Salt Solution (HBSS; pH 7.4) was slowly instilled into the lung until fully inflated. Immediately after agarose inflation, the lungs were washed with ice-cold HBSS, packed with ice and allowed to cool at 4 °C for 30–45 min. The lung lobe was sectioned into slices 150 μm thick using Electron Microscopy Sciences Tissue slicer (OTS 4500).

Sections of the lung were then transferred to wells of a 24-well plate and maintained with DMEM supplemented with 10% FBS, antibiotics, and antimycotics at 37 °C and 10% CO_2_ for at least 5 days prior to treatment. Twenty-four hours prior to treatment, media was changed to serum free media. Lung slices were then treated with SPD-MAA for 24 h. After 24 h the supernatant was collected and stored at −80 °C for further chemokine analysis. Lung slices from each treatment were collected and homogenized in cell lysis buffer and the protein concentration (mg/mL) was measured using the Bradford assay [32]. The chemokine level was normalized to the concentration of protein.

### Real-time Quantitative RT-PCR (qRT-PCR)

RNA was isolated from the lung tissue using RNeasy mini kit (Qiagen, Valencia, CA) following manufacturer instructions as previously described [33, 34]. After RNA isolation, the concentration and purity of RNA was determined by NanoDrop spectrophotometer. TaqMan reverse transcription kit (Applied Biosystems, Branchburg, NJ) was used to synthesize DNA from 100 ng of template RNA purified from lung tissue. Real-time quantitative PCR was performed on the cDNA using the following reaction: 1× TaqMan master mix and mouse CD204 primer (Applied Biosystems, Branchburg, NJ; Mm00446214_m1) and probe mix in 25-μl reactions in a 96-well plate in duplicate. The plate was placed in an ABI Prism 7500 Sequence detection system (Applied Biosystems). Reactions were carried out for 2 min at 50 °C, 10 min at 95 °C, then 40 cycles of 15 s at 95 °C and 1 min at 60 °C. Data are reported as fold-change from control.

### SR-A1 ELISA

For measurement of SR-A1 expression on whole lung tissue, the protein was isolated from the lung tissue. The lung tissue was rinsed in PBS to remove excess blood. The tissue was then homogenized in 500 μl of PBS at 4 °C. The homogenate was then centrifuged at 15,000 g for 10 mins. After centrifugation, the supernatant was collected and protein was measured using the Bradford assay. The supernatant was used to perform the sandwich ELISA according to manufacturer’s instructions (LifeSpan Bioscience Inc, Seattle, WA).

### Statistical analyses

All data were analyzed using Graphpad Prism 5 (San Diego, CA, USA). Results represent mean ± standard error. One- and two-way ANOVA with Tukey post-hoc test and one-sample t-test were used to analyze data for statistical significance. *P* < 0.05 was accepted as statistically significant.

## Results

### SR-A1 KO mice showed decreased lung cellularity following repetitive MAA instillation

**Fig. 1:**
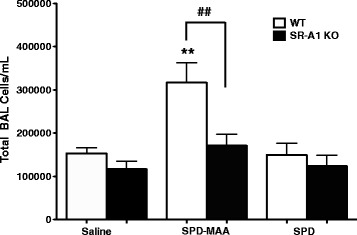
Total cell count in BALF after 3 weeks of intranasal instillation of SPD-MAA. Total cell count in BALF from WT and SR-A1 KO mice after intranasal instillation of saline, SPD-MAA (50 μg/mL) and SPD (50 μg/mL) for 3 weeks. Values are presented as the mean ± SEM, n = 6-10 mice/group. **P < .01 for saline vs SPD-MAA. ## *P* < .01 for WT vs SR-A1 KO

Repetitive instillation of SPD-MAA has been previously reported to increase cellular influx in lavage fluid in WT mice. When SR-A1 KO mice were repeatedly instilled with SPD-MAA (50 μg/mL) for 3 weeks, however, a significant decrease (*p* < 0.01) in total lung lavage cells was observed when compared to WT mice (Fig. 1). No difference was observed in both WT and SR-A1 KO mice instilled with saline or non-adducted SPD. These data suggest that SR-A1 could be important in SPD-MAA mediated effects on lung cellularity. This result also suggests that SPD-MAA mediated increases in total BAL cellularity is SR-A1 dependent.

### SR-A1 KO mice showed decreased neutrophil influx following repetitive MAA instillation

**Fig. 2:**
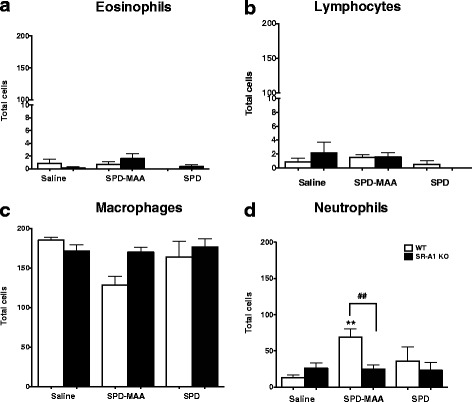
Differential white blood cell counts in BALF collected after intranasal administration of SPD-MAA, SPD or saline. Differential cell eosinophils **a** lymphocytes **b** macrophages **c** and neutrophils **d** count in BALF from WT and SR-A1 KO mice after intranasal instillation of saline, SPD-MAA (50 μg/mL) and SPD (50 μg/mL) for 3 weeks. Values are presented as the mean ± SEM, *n* = 6–10 mice/group. **P < .01 for saline vs SPD-MAA. ## *P* < .01 for WT vs SR-A1 KO

Repeated instillation for 3 weeks of 50 μg/mL SPD-MAA resulted in an increased influx of neutrophils in lung when compared to the saline-instilled group (Fig. 2d). There were no changes in eosinophils (Fig. 2a) or lymphocytes (Fig. 2b). When SR-A1 KO mice were repeatedly instilled with SPD-MAA, significantly (p < 0.01) fewer neutrophils were observed when compared to SPD-MAA instilled WT mice (Fig. 2d). Likewise, the seemingly decrease in macrophages seen in WT mice after MAA instillation compared to WT saline (Fig. 2c) is a result of the increased neutrophils represented in the total 200 cells counted for each treatment group. Saline and non–adducted SPD instillation have no effect on the neutrophil or macrophage count in the BAL fluid. These results suggest that the decrease in total lung cellularity observed after SPD-MAA instillation in SR-A1 KO mice was due to a decreased influx of neutrophils compared to control. These results also suggest that SR-A1 is important for MAA-mediated neutrophil recruitment.

### MAA adduct-stimulated chemokine release is SR-A1-dependent in vitro

**Fig. 3:**
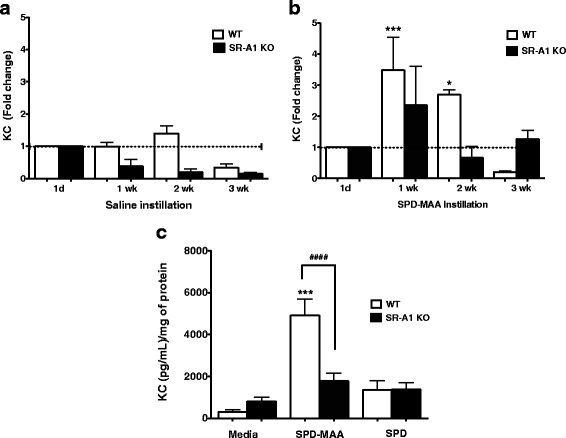
Neutrophil chemokine KC (CXCL1) in BAL and from lung slices. KC levels in BAL samples of mice instilled with saline **a** or SPD-MAA: 50 μg/mL **b** were measured over 1d, 1–3 weeks time. Lung slices from both WT and SR-A1 KO mice were treated with media, SPD-MAA (100 μg/mL) and non-adducted SPD (100 μg/mL) for 24 h **c**. After 24 h supernatant was collected and neutrophil chemokine KC release was measured. Values are presented as the mean ± SEM of *n* = 3 independent experiments for lung slice and *n* = 5–16 mice per group for BAL KC release. ***P < .001 and *P < .05 for 1 and 2 weeks vs 1d instillation. *** *P* < .001 for media vs SPD-MAA. #### *P* < .0001 for WT vs SR-A1 KO

To determine the role of SR-A1 in MAA adduct-stimulated KC levels in BAL samples from mice instilled for 1 day, 1 week, 2 weeks or 3 weeks was also measured. No increase in KC release was observed over any of the time periods in saline-instilled WT mice (Fig. 3a). In WT SPD-MAA instillation group, there was a 3.5 fold (215.9 ± 65 pg) increase in KC release in the 1-week (*p* < 0.001) and 2.7 fold (167.2 ± 8.944 pg) increase in 2-week (*p* < 0.05) mice when compared to the 1-day animals (61 ± 16.98 pg) (Fig. 3b). At 3 weeks, KC release returned to baseline (Fig. 3b). In SR-A1 KO mice there was no significant increase in KC release over time in both saline-instilled (Fig. 3a) and SPD-MAA instilled mice (Fig. 3b). In support of these observations, ex vivo treatment with SPD-MAA resulted in a significant increase (p < 0.001) in KC release from lung slices made from WT mice when compared to media control treatment (Fig. 3c). In contrast, a significant reduction (*p* < 0.0001) in KC release was observed from lung slices of SR-A1 KO mice when compared with WT lung slices treated with MAA (Fig. 3c). No increase in KC release was observed over the time period of SPD instillation in WT mice (data not shown). These data show that SR-A1 is required for MAA-stimulated chemokine release in an *in vitro* lung slice model.

### Repeated exposures to MAA adduct increased whole lung SR-A1 expression

**Fig. 4:**
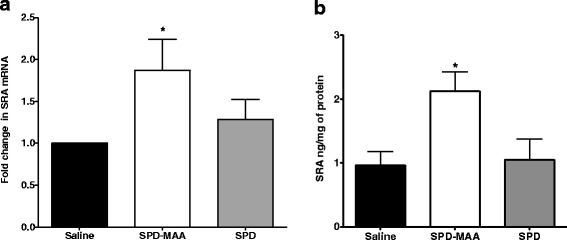
mRNA and protein levels of SR-A1 (CD204) in total lung tissue from WT mice. mRNA **a** and protein **b** levels of SR-A1 on whole lung tissue after 3-week saline, SPD-MAA (50 μg/mL) and SPD (50 μg/mL) instillation. Values are presented as the mean ± SEM, n = 5 mice/group. *P < .05 for saline vs SPD-MAA

To determine the role of MAA adduct on lung SR-A1 (CD204) expression, lung tissue from WT mice instilled with 50 μg/mL SPD-MAA for 3 weeks was used to measure SR-A1 message and protein expression. Repeated MAA exposure resulted in significant increase (*p* < 0.05) in SR-A1 message expression (almost twofold; Fig. 4a). Consistent with the message expression, a significant increase (*p* < 0.05) in SR-A1 protein expression was also observed (Fig. 4b). No such increase in both SR-A1 message and protein was seen in WT mice instilled with non-adducted SPD (Fig. 4a, b). These data may be important in explaining the increased inflammation in WT mice due to repeated exposure to MAA adduct.

### SR-A1 KO mice display reduced lung inflammation following repeated MAA adduct instillation

**Fig. 5:**
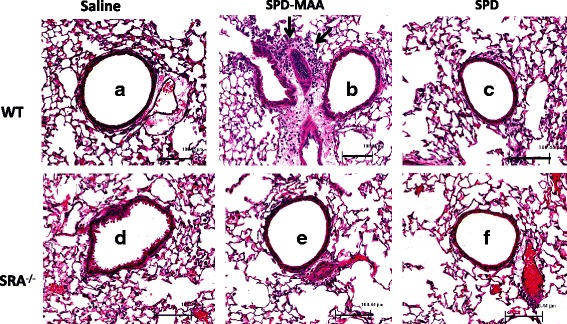
Lung inflammation in lung after 3 weeks intranasal instillation of SPD-MAA. Both WT and SR-A1 KO mice were treated intranasally with saline, SPD-MAA (50 μg/mL) and SPD (50 μg/mL) for 3 weeks. A representative 4–5 μm thick section of H and E stained of one mouse per treatment group is shown (10 × magnification). WT Saline **a**, SPD-MAA **b**, SPD **c** and SR-A1 KO Saline **d**, SPD-MAA (E), SPD **f**. Line scale represents approx. 100 μm. Arrow denotes the localization of inflammatory cells

Our previous study shows that repetitive exposures to MAA adduct results in lung inflammation due to the influx of inflammatory cells within the peri-bronchiolar region of small airways [20]. To determine the role of SR-A1 on such inflammation, SR-A1 KO mice were instilled with 50 μg/mL of SPD-MAA adduct for 3 weeks and later paraffin-embedded whole lungs were sectioned and stained for hematoxylin and eosin. In comparison to WT mice (Fig. 5b), microscopic examination revealed significantly fewer inflammatory cells within the peri-bronchiolar region of the small airways of SR-A1 KO mice exposed to MAA adduct (Fig. 5e). No such change (influx of neutrophils) was observed in the lungs of mice instilled with either saline (Fig. 5a, d) or non-adducted SPD (Fig. 5c, f) for both strains of mice.

### SR-A1 KO mice display reduced immunoreactivity for MAA adduct after repeated exposure

**Fig. 6:**
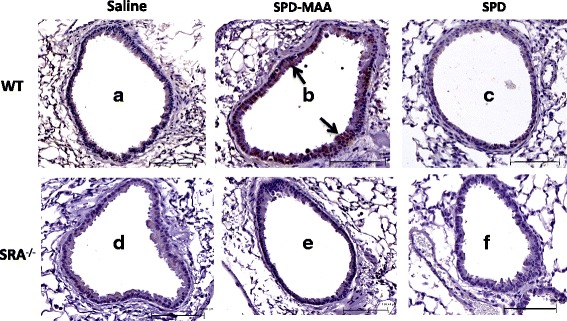
Representative lung tissue sections immunohistochemically stained for SPD-MAA. Immunohistochemical staining of SPD-MAA in lung airways of both WT and SR-A1 KO mice treated intranasally with saline, SPD-MAA (50 μg/mL) and SPD (50 μg/mL) for 3 weeks. A representative 4–5 μm-thick section of one mouse per treatment group is shown (20 × magnification). WT Saline **a** WT SPD-MAA **b** WT SPD **c** and SR-A1 KO Saline **d**, SR-A1 KO SPD-MAA **e**, SR-A1 KO SPD **f**. Line scale represents approx. 100 μm. Arrow denotes the staining and binding of MAA adduct

Previous studies show that SR-A1 is important for MAA adduct binding in lung epithelial cells [29]. To further investigate that the decreased MAA-mediated effect in SR-A1 KO mice could be due to decreased binding of MAA adduct in lung tissue after 3-week instillation, whole lung sections from both WT and SR-A1 KO mice were stained for MAA adduct. Immunohistochemical staining for MAA adduct demonstrated increased immunoreactivity along the bronchial epithelial and columnar epithelial cells around the airways in WT mice (Fig. 6b). No staining was observed in WT and SR-A1 KO mice instilled with saline (Fig. 6a, d) or non-adducted SPD (Fig. 6c, f). No staining was observed with isotype control antibody (data not shown). These results suggest that SR-A1 is important for MAA binding in airway as no such staining was detected in KO mice. This could further explain the decreased response to MAA adduct in SR-A1 KO mice.

## Discussion

In this study, we demonstrated for the first time the mechanistic role SR-A1 plays in regulating previously reported MAA adduct-induced lung inflammation and lung injury. Repetitive instillation of MAA adduct in mouse lung resulted in inflammation as a result of neutrophil influx in the peri-bronchial region. Increased lung cellularity and increased influx of neutrophils were observed in the lungs of WT mice, but in SR-A1 KO mice all of these effects caused by MAA adduct after repetitive instillation were significantly diminished. This diminished inflammation in SR-A1 KO mice could be further explained by a decrease in MAA adduct binding to the airway cells. As a result, a decrease in KC release from lung slices was observed. Similarly, no significant increase in KC release in BAL from SR-A1 KO mice instilled with SPD-MAA at 1 and 2 weeks was observed. This could explain the decreased neutrophil influx in the lung of the SR-A1 KO mice after MAA adduct instillation. An increase in KC release in BAL from WT mice instilled for 1 and 2 weeks suggests earlier KC release, leading to the recruitment of neutrophils in the airways at 3 weeks. Our study is the first one to show the importance of SR-A1 in MAA-induced lung inflammation in mouse lung.

Previous studies show that macrophages scavenge chemically modified proteins such as formaldehyde-treated bovine serum albumin, maleylated albumin and malondialdehyde-modified and acetylated low-density lipoprotein by endocyting via a receptor mediated mechanism [35]. Highly reactive aldehydes formed as a result of smoking and drinking have the ability to modify lung proteins to produce a highly stable and immunogenic product, malondialdehyde-acetaldehyde (MAA) adduct [36, 37]. In contrast to individual protein adducts formed by AA and MDA, which are unstable and dissociate rapidly, this MAA adduct is stable [38, 39] and can remain in the lung for a long time. In mouse lung, surfactant proteins A (SPA) and D (SPD) are equally MAA-adducted when exposed to both ethanol and cigarette smoke [20]. This suggests that surfactant proteins are ideal targets for MAA adduction in lung and therefore SPD-MAA used in the study would be relevant to human subjects who abuse alcohol and smoke cigarettes.

The presence of MAA adduct in liver, lung and recently in the rheumatoid arthritis synovial tissue suggests its importance as a marker of inflammation [40]. In lung, SR-A1 expressed on bronchial epithelial cells is reported to bind MAA adduct and internalize them [29]. In support of this result, we earlier reported that SR-A1 was involved in MAA-adducted protein-stimulated, PKC/ε-mediated KC production in mouse airway epithelial cells [29]. We showed that MAA adduct binding to bronchial epithelial cells is blocked when pretreated with fucoidan, a ligand for scavenger receptor A [19]. Additionally, a decreased antibody response to MAA-Alb in SR-A1 KO mice is also reported [30]. Because SR-A1 is extensively expressed in macrophages, a similar result was also reported when macrophages from SR-A1 KO mice were treated with MAA adduct. A decrease in the pro-inflammatory cytokines, TNF alpha and IL-6, was observed when compared to macrophages from WT mice [18]. Willis et al. also reports that MAA-haptenated proteins are preferentially bound by scavenger receptors on macrophages, which internalize the ligands and shuttle them to lysosomes [41]. Our result further supports these published results that SR-A1 is one of major receptors involved in the effects mediated by MAA-modified proteins in mouse lung.

In the absence of SR-A1, we observed no accumulation of MAA adducted protein in the lungs of the KO mice, unlike the binding of SPD-MAA to epithelial cells and macrophages of the lung in wild type mice. Secreted surfactant has three different fates: (a) recycling, governed by the alveolar type II cell, followed by re-secretion; (b) degradation followed by the synthesis of new surfactant protein; and/or (c) removal from the surfactant system [42]. Constitutive mucociliary clearance serves as the major whole lung removal mechanism for surfactant. Cleared surfactant is swallowed into the gastrointestinal tract and eventually excreted. Thus, the likely outcome for unbound MAA-SPD in KO mice (and to some extent in wild type mice as well) would be the normative process of lung clearance.

There is a similarity between the mechanism by which both oxidized LDL (ox-LDL) and modified MAA protein initiate toxicity [43]. Both are recognized by scavenger receptors and Ox-LDL is associated with the pathogenesis of atherosclerosis [44]. There are also various theories suggesting the specific feature of the ligand that make it suitable for the scavenger receptor. Reaction of amino groups of protein with short-chain α-hydroxy aldehydes may yield a moiety that is important for recognition by scavenger receptor A [45]. Negative charges in the ligands could be another important feature for bonding reaction [46]. Change in protein confirmation after binding with aldehydes could be another determinant in receptor binding [47]. MAA adduct formed as a result of Schiff base reaction between 2 mol of malondialdehyde and 1 mol of acetaldehyde with lung protein, such as SPD, therefore, may fulfill the above-mentioned requirements, making it a ligand for SR-A1.

Our study also showed a difference in MAA staining in the lung after repeated intranasal instillation for 3 weeks in WT and SR-A1 KO mice. Interestingly, most of the positive staining for MAA was seen in airway epithelial cells, the cells that first come into contact with MAA after intranasal instillation. Airway epithelial cells express SR-A1 and take up MAA as previously reported [29]. The remarkable finding of our study was that in SR-A1 KO mice, significantly less MAA staining was observed. This further supports the observation that the diminished response to MAA in SR-A1 KO mice is due to less uptake of MAA by airway epithelial cells. Our results also suggest that binding of MAA to airway epithelial cells is important to initiate the inflammatory responses to MAA.

Our study also demonstrated an increase in SR-A1 expression after repetitive instillation of MAA adduct for 3 weeks. The increased inflammation in the lung of WT mice after MAA instillation could be due to increased binding as a result of increased expression of the SR-A1 on the lung. This modulation of SR-A1 expression in lung is important as it could modulate the inflammatory responses to MAA adduct. Chronic lung inflammation is one of the characteristic features of COPD, which is predicted to be the third leading cause of death worldwide by 2020 [48]. Smoking is the primary risk factor for COPD [49] and about one-third of smokers are drinkers. This is why it is important to study the role of co-exposure of cigarette smoke and alcohol on lung inflammation. One of the pathways through which this co-exposure could induce lung injury is the formation of MAA adduct from reactive aldehydes. Our results suggest that SR-A1 is important to initiate MAA-mediated inflammation in lung. Previous studies have also reported that genetic polymorphisms in macrophage scavenger receptor-1 gene (MSR1) could result in increased macrophage adhesion, receptor expression, and reduced apoptosis [50]. It has been also linked to COPD susceptibility [51].

## Conclusions

In summary, this study demonstrates the functional role of SR-A1 in MAA adduct-induced inflammation in lung. SR-A1 is important for initiation of inflammation after instillation of MAA as absence of SR-A1 is protective against MAA mediated lung inflammation and injury. Future studies involving individuals with genetic polymorphisms in SR-A1 would be important to better understand the role of SR-A1 on susceptibility to lung inflammation in smokers and drinkers.

## Abbreviations

AA: Acetaldehyde
ADH: Alcohol dehydrogenase
ARDS: Acute respiratory distress syndrome
AUDs: Alcohol use disorders
BAL: Bronchoalveolar lavage
COPD: Chronic obstructive pulmonary disease
H&E: Hematoxylin and eosin
KC: keratinocyte chemoattractant
MAA: Malondialdehyde-acetaldehyde
MAA-Alb: Malondialdehyde acetaldehyde albumin
MDA: Malondialdehyde
MSR1: Macrophage scavenger receptor-1 gene
ox-LDL: Oxidized LDL
qRT-PCR: Real-time quantitative RT-PCR
SPD: Surfactant protein D
SPD-MAA: SPD adducted malondialdehyde-acetaldehyde adduct
SR-A1: Scavenger receptor A
